# Child mortality from sickle cell disease in Nigeria: a model-estimated, population-level analysis of data from the 2018 Demographic and Health Survey

**DOI:** 10.1016/S2352-3026(21)00216-7

**Published:** 2021-09-02

**Authors:** Obiageli E Nnodu, Assaf P Oron, Alayo Sopekan, Godwin O Akaba, Frédéric B Piel, Dennis L Chao

**Affiliations:** a Centre of Excellence for Sickle Cell Disease Research and Training, University of Abuja, Abuja, Nigeria; b Institute for Disease Modeling, Bill & Melinda Gates Foundation, Seattle, WA, USA; c Sickle Cell Disease Desk, Noncommunicable Diseases Control Programme, Department of Public Health, Federal Ministry of Health, Abuja, Nigeria; d Department of Obstetrics and Gynaecology, College of Health Sciences, University of Abuja, Abuja, Nigeria; e Department of Epidemiology and Biostatistics, School of Public Health, Imperial College London, London, UK

## Abstract

**Background:**

Child mortality from sickle cell disease in sub-Saharan Africa is presumed to be high but is not well quantified. This uncertainty contributes to the neglect of sickle cell disease and delays the prioritisation of interventions. In this study, we estimated the mortality of children in Nigeria with sickle cell disease, and the proportion of national under-5 mortality attributable to sickle cell disease.

**Methods:**

We did a model-estimated, population-level analysis of data from Nigeria's 2018 Demographic and Health Survey (DHS) to estimate the prevalence and geographical distribution of *HbSS* and *HbSC* genotypes assuming Hardy-Weinberg equilibrium near birth. Interviews for the survey were done between Aug 14 and Dec 29, 2018, and the embedded sickle cell disease survey was done in a randomly selected third of the overall survey's households. We developed an approach for estimating child mortality from sickle cell disease by combining information on tested children and their untested siblings. Tested children were aged 6–59 months at the time of the survey. Untested siblings born 0–14 years before the survey were also included in analyses. Testing as part of the DHS was done without regard to disease status. We analysed mortality differences using the inheritance-derived genotypic distribution of untested siblings older than the tested cohort, enabling us to estimate excess mortality from sickle cell disease for the older-sibling cohort (ie, those born between 2003 and 2013).

**Findings:**

We analysed test results for 11 186 children aged 6–59 months from 7411 households in Nigeria. The estimated average birth prevalence of *HbSS* was 1·21% (95% CI 1·09–1·37) and was 0·24% (0·19–0·31) for *HbSC*. We obtained data for estimating child mortality from 10 195 tested children (who could be matched to the individual mother survey) and 17 205 of their untested siblings. 15 227 of the siblings were in the older-sibling cohort. The group of children with sickle cell disease born between 2003 and 2013 with at least one younger sibling in the survey had about 370 excess under-5 deaths per 1000 livebirths (95% CI 150–580; p=0·0008) than children with *HbAA*. The estimated national average under-5 mortality for children with sickle cell disease born between 2003 and 2013 was 490 per 1000 livebirths (95% CI 270–700), 4·0 times higher (95% CI 2·1–6·0) than children with *HbAA*. About 4·2% (95% CI 1·7–6·9) of national under-5 mortality was attributable to excess mortality from sickle cell disease.

**Interpretation:**

The burden of child mortality from sickle cell disease in Nigeria continues to be disproportionately higher than the burden of mortality of children without sickle cell disease. Most of these deaths could be prevented if adequate resources were allocated and available focused interventions were implemented. The methods developed in this study could be used to estimate the burden of sickle cell disease elsewhere in Africa and south Asia.

**Funding:**

Sickle Pan African Research Consortium, and the Bill & Melinda Gates Foundation.

## Introduction

Sickle cell disease is caused by a mutation in the β-globin gene (*HBB*) that leads to the production of an abnormal form of the β-subunit of haemoglobin, HbS.^1^ It is the most common life-threatening genetic disorder among people of African ancestry. Without health care targeted towards sickle cell disease, most children born with the more severe forms of the disease, such as sickle cell anaemia, which accounts for most sickle cell disease births, would not survive to adulthood.^2^ The progress made since 2000 in high-income and middle-income countries has shown that a suite of interventions anchored by the screening of newborn babies can improve the probability of survival for children with sickle cell disease to adulthood to the same level as the general population.^3^, ^4^, ^5^

In sub-Saharan Africa, where most births of babies with sickle cell disease take place,^6^ progress has been far slower than in high-income and middle-income countries. Mobilisation of resources to address sickle cell disease in sub-Saharan Africa are hampered, in part, by an absence of agreed-upon health burden estimates. Most public statements about mortality from the disease in this region are derived from a review of published evidence,^7^ suggesting that under-5 mortality of children with sickle cell disease in sub-Saharan Africa is 50–90%. In many sub-Saharan African countries, this sickle cell disease-associated mortality estimate would translate to at least 5–10% of overall under-5 mortality. By contrast, the 2019 Global Burden of Disease (GBD) Study^8^ attributed to sickle cell disease only 0·66% (95% CI 0·41–0·94) of general under-5 mortality in sub-Saharan Africa. In Nigeria the attribution was 0·82% (95% CI 0·37–1·34). Discrepancies such as these contribute to the persistent neglect of global attention to child mortality from sickle cell disease in sub-Saharan Africa.

**Research in context**

**Evidence before this study**

We did not do a formal systematic review. A previous review of the scientific literature found consistent reports of 50–90% early-life mortality among children born in Africa with sickle cell anaemia (*HbSS*). A prospective cohort study in Kilifi, Kenya, noted child mortality from sickle cell anaemia of 58% (95% CI 40–86) per 1000 person-years of observation, comprising 15% of the cohort's under-5 deaths. By contrast, the Global Burden of Disease Study attributes only 0·66% (95% CI 0·41–0·94) of under-5 deaths in sub-Saharan Africa to sickle cell disease.

**Added value of this study**

Our study showed the benefit of testing for sickle cell disease in national health surveys and describes a new method for estimating mortality from sickle cell disease from such surveys. We found that child mortality from sickle cell disease is high in Nigeria and contributes to a substantial portion of the country's under-5 mortality. To our knowledge, this is the first estimate of child mortality from sickle cell disease based on nationally representative data from a sub-Saharan African country.

**Implications of all the available evidence**

Our study strengthens existing evidence that the health burden of sickle cell disease and its contribution to under-5 child mortality is still disproportionately high and is often underestimated. More accurate estimates might enable effective targeting of resource allocations and implementation of cost-effective interventions, particularly in regions with a high prevalence of sickle cell disease, such as sub-Saharan Africa and south Asia.

One major challenge to quantifying the health burden of sickle cell disease has been the absence of diagnosis for most children with the disease in sub-Saharan Africa. Newborn and early-infancy screening is rare, leading to misattribution of many deaths related to sickle cell disease. However, there have been several promising developments over the past 7 years. The testing of nearly 100 000 dried-blood spots collected for HIV screening has produced detailed maps of Uganda's early-childhood prevalence of sickle cell disease.^9^ Additionally, new, reliable, and affordable point-of-care testing devices have been developed and are included in WHO's latest Essential Diagnostic List.^10^ Several studies, including some from Nigeria, have shown the reliability and feasibility of point-of-care testing kits for sickle cell disease screening in sub-Saharan Africa.^11^

Nigeria, Africa's most populous country with a population of 206 million, is probably the country with the world's largest population of individuals with sickle cell disease. Health-care access is often inadequate, with the costs of health care borne by individuals. Health insurance coverage is about 3% and is employee based.^12^ Sickle cell disease is recognised as a priority non-communicable disease and the Nigerian Government developed guidelines for its prevention and control in 2013, as well as a policy and protocol for newborn screening.^13^ The government established six comprehensive newborn screening centres in each of the geopolitical zones of the country, but the high cost of diagnostic reagents and inadequately trained personnel have precluded full functioning of these centres.^14^

The 2018 Nigeria Demographic and Health Survey (DHS)^12^ was the first national health survey to include data on sickle cell disease. By combining point-of-care testing for sickle cell disease with a standard nationally representative sampling of child health and survival in the general population, it offers an excellent opportunity to refine mortality estimates for the disease. Using this unique dataset, our aims for this study were to estimate national and subnational prevalence of sickle cell disease and compare it to previous estimates, and to derive a quantitative estimate of child mortality from sickle cell disease in Nigeria.

## Methods

### Data source

We used data from Nigeria's 2018 DHS, which used a stratified, two-stage sample design with 74 strata in the first stage and about 1400 clusters in the second stage. Interviews were done from Aug 14 to Dec 29, 2018.^12^ The embedded survey module on sickle cell disease was carried out in a randomly selected third of the overall survey's households (ie, 30 households were selected per cluster using equal probability systematic sampling of households, and a third of these were selected for biomarker collection). In these households, children aged 6–59 months considered de-facto residents (ie, they had slept in that household on the previous night) were screened for *HBB* alleles using SickleSCAN point-of-care testing devices (BioMedomics, Research Triangle Park, NC, USA). The DHS also collected standard child morbidity and mortality data in its individual (maternal) module. We obtained birth cohort size estimates from WorldPop's 1 km × 1 km raster birth estimates for Nigeria in 2015.^15^ We used shapefiles from the database of Global Administrative Areas^16^ to derive state-level estimates from WorldPop's raster. A drop of blood from a finger or heel prick was obtained by a field team at the home and tested immediately using a SickleSCAN kit. Consent for obtaining and testing children's blood samples was given by a parent or adult responsible for the child. Data collection procedures were approved by the National Health Research Ethics Committee of Nigeria and the ICF Institutional Review Board. Further details are in the DHS final report.^12^ Approval to use the data^17^ was obtained from the DHS programme.

### Statistical analysis

We estimated *HbSS* and *HbSC* prevalence from allele frequencies, assuming Hardy-Weinberg equilibrium near birth. Although selection pressures and assortative mating probably keep the *HBB* allele distribution out of equilibrium,^18^ we consider Hardy-Weinberg equilibrium as a practical way to stabilise and reduce bias in estimating the sickle cell disease genotypes, leveraging sickle trait counts that are more numerous and presumably less susceptible to early attrition due to child mortality. The observed frequency of *HbSS* at the national, regional, and state levels is compared with that expected assuming Hardy-Weinberg equilibrium (appendix p 9). These calculations were done for Nigeria's six geopolitical zones. We derived *A, S*, and *C* allele frequencies from observed genotype frequencies using household sample weights. The DHS data format did not include the option of *HbCC* genotype. In our *C* allele prevalence estimates we assumed that the nine “other” entries were *HbCC*, since they all occurred in zones with a relatively high prevalence of *HbC*.

We used bootstrap sampling to estimate uncertainty in strata-level allele frequencies. For each DHS sampling stratum, clusters in which at least one child was tested were sampled with replacement. This process was repeated 1000 times to derive the 95% CIs. By bootstrap sampling of clusters instead of individuals or households, clustering of children within households and households within villages were taken into account.^19^

**Figure 1:**
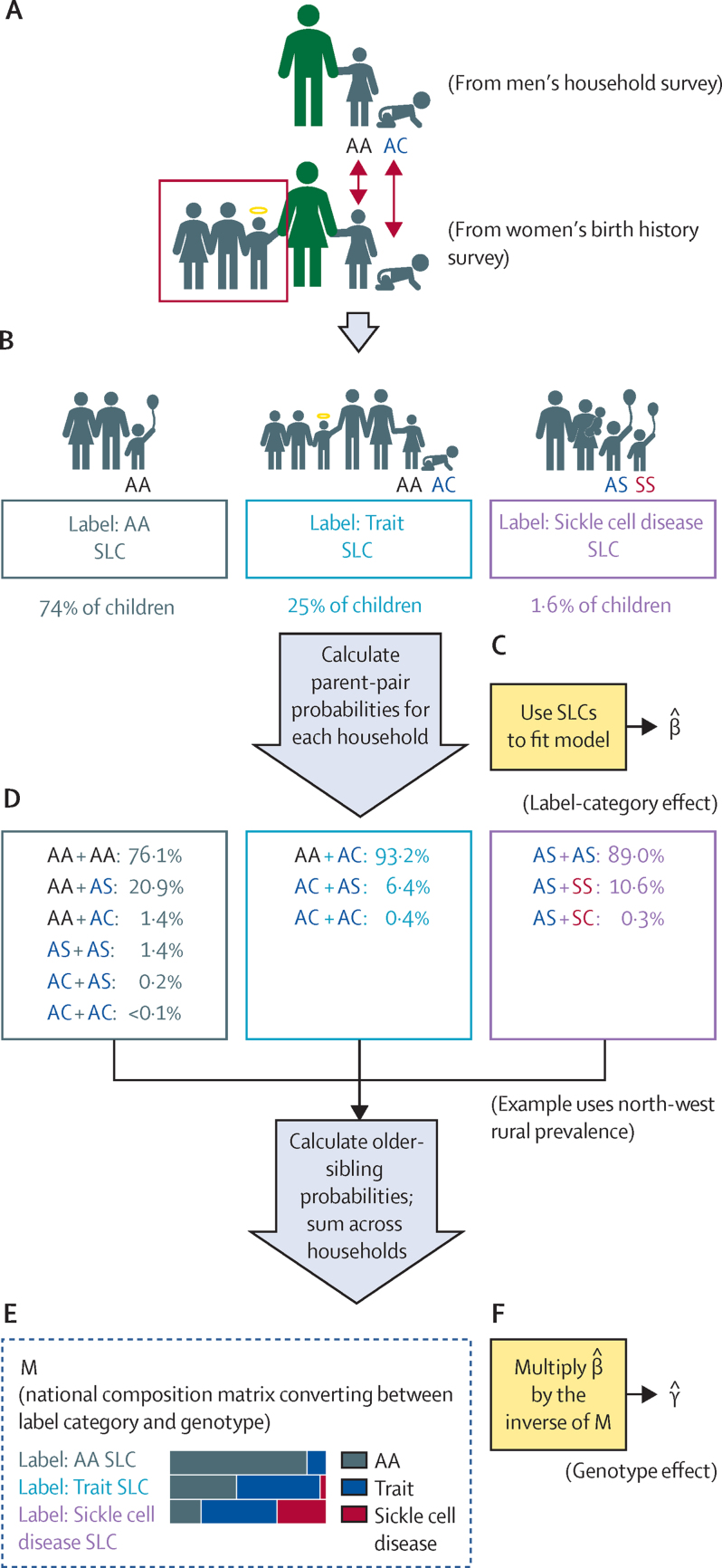
Schematic of the sibling-group labelling and excess mortality analysis Normal *HBB* genotype (*AA*) is shown in black, sickle trait genotypes (*AS* and *AC*) in blue, and sickle cell disease genotypes (*SS* and *SC*) in red. (A) Children were tested for *HBB* genotype in the household survey. The green male (father) figure illustrates household identification. Household and maternal survey modules are merged, combining sickle cell disease information from the household survey module and birth histories from the maternal survey module and identifying siblings. The green female figure is the mother, who was interviewed for the birth history survey. The yellow halo represents a deceased sibling. (B) Every sibling group received a label as explained in the Methods (AA SLC, Trait SLC, or Sickle cell disease SLC). (C) We used regression models to estimate excess under-5 mortality between SLCs; excess under-5 mortality is denoted by β in the figure. (D) Parent-pair posterior probabilities were calculated, and from them older-sibling probabilities, which were then summed across all households in each SLC. (E) Summing of older-sibling probabilities produced M, a matrix with the expected overall genotype composition of each SLC. (F) Multiplying β by the inverse of M produced an estimate of relative genotype survival, γ. SLC= sibling-group label category.

To estimate excess mortality, we first identified tested children and their sibling, then categorised each sibling group according to the available information on sickle cell disease. Paternal siblings were implicitly identified by living in the same household (figure 1). Because of shared parentage, untested siblings of tested children with sickle cell disease are far more likely to have the disease than children without siblings with known sickle cell disease. To identify maternal siblings both alive and deceased, the children's mothers were identified in the individual (mothers') interviews using child identifiers. Children living in the same household and sharing the same mother identifiers were assumed to be full siblings, and those only sharing either household identifiers or mother identifiers were considered half-siblings. For this part of the analysis, entries with other genotypes were excluded, but all other test data were included, regardless of tested child age and de-facto residence.

For each group of full siblings, all available genotypes were pooled, and the group was labelled by its most severe *HBB* genotype, according to the ordering Sickle cell disease (*SS*/*SC*) > Trait (*AS*/*AC*) > AA (figure 1). This process divided the dataset into three sibling-group label categories (SLCs). Children with tested half-siblings but no tested full siblings were given the label of their half-siblings, unless that label was Sickle cell disease, in which case they received the label Trait SLC. Similarly, sibling groups labelled AA SLC on the basis of full siblings were relabelled Trait SLC if they had half-siblings labelled Sickle cell disease. Descriptive non-parametric Kaplan-Meier curves for the three SLCs through to age 5 years for children born 0–14 years before the survey were calculated and plotted, stratified by geopolitical zone and combined for a single national curve.

Our main analysis to estimate the genotype effect started with a linear regression model of the association between sibling-group label category and excess under-5 mortality among older siblings of tested children, born 5–14 years before the survey. The choice of linear regression was based on synthetic-data simulations revealing it to be unbiased, more precise, and more robust than logistic regression, when used in combination with the analysis method described later. This model adjusted for Nigeria's six geopolitical regions and urban or rural residence, and included a random intercept for the survey cluster.

We developed an approach to estimate the relative mortality of children with sickle cell disease or sickle trait with respect to *HbAA*. Differences in sibling-group label category survival can be driven by the latent variable of true child genotype. We applied an empirical Bayesian linear decomposition approach to the sibling-group label category model for children born 5–14 years before the survey, to derive mortality estimates for each genotype. Because of the monogenic nature of sickle cell disease, we could derive a posterior probability for the parents of tested children, and from them for non-tested full siblings as well (figure 1). This was only appropriate to age groups not included in the survey, because in the 6–59 months group the most common reason for not being tested was the child's death. The approach is described in further detail in the appendix (pp 2–4).

We calculated the resulting model-estimated population-level child mortality rates from sickle cell disease, and the proportion of subnational and national child mortality attributable to sickle cell disease. To obtain rough estimates of absolute mortality burden, we used official UNICEF estimates for under-5 deaths in Nigeria during the relevant period. We used R version 4.0 for data analysis.

### Role of the funding source

The funders of the study had no role in study design, data collection, data analysis, data interpretation, or writing of the report.

## Results

In the 2018 Nigeria DHS, 11 386 children aged 6–59 months were eligible, and we recorded test results for 11 186 children from 7411 households. Of these children, when sample weights were applied, 77·2% were found to have *HbAA*, 19·7% had *HbAS*, 1·6% had *HbAC*, 1·3% had either *HbSS* or *HbSC*, and 0·1% were recorded as other (appendix p 11). All six geopolitical zones of Nigeria had at least a 10% prevalence of the *S* allele, whereas *C* allele prevalence was highest in the south-west zone and nearly absent in the eastern part of the country (table 1). *S* allele frequency differed between rural and urban populations by state, but not in a consistent direction (appendix p 7).

**Table 1:** Observed allele frequencies in Nigeria by geopolitical zone

	**A allele**	**S allele**	**C allele**	**Sickle cell disease (genotypes)**	
				*HbSS*	*HbSC*
North central	88·9% (87·6–90·1)	10·0% (8·9–11·2)	1·1% (0·7–1·4)	0·93% (0·51–1·44)	0·36% (0·13–0·65)
North east	88·4% (87·0–89·8)	11·3% (10·0–12·7)	0·3% (0·1–0·6)	0·90% (0·44–1·44)	0·26% (0·00–0·74)
North west	88·2% (87·1–89·3)	11·1% (10·0–12·2)	0·7% (0·4–1·0)	1·00% (0·60–1·50)	0·15% (0·02–0·33)
South east	89·3% (88·2–90·5)	10·6% (9·5–11·7)	0·1% (0·0–0·2)	1·00% (0·55–1·57)	0·12% (0·00–0·35)
South south	90·0% (88·6–91·3)	9·9% (8·611·1)	0·1% (0·0–0·3)	0·32% (0·09–0·63)	0·00% (0·00–0·00)
South west	84·5% (81·7–86·3)	12·1% (10·5–14·3)	3·4% (2·6–4·3)	0·82% (0·41–1·32)	1·52% (0·59–3·12)
Nigeria	88·0% (87·3–88·6)	11·0% (10·4–11·6)	1·0% (0·9–1·3)	0·88% (0·69–1·10)	0·43% (0·23–0·74)

Data are medians (95% CIs) derived from 1000 bootstrap samples.

**Figure 2:**
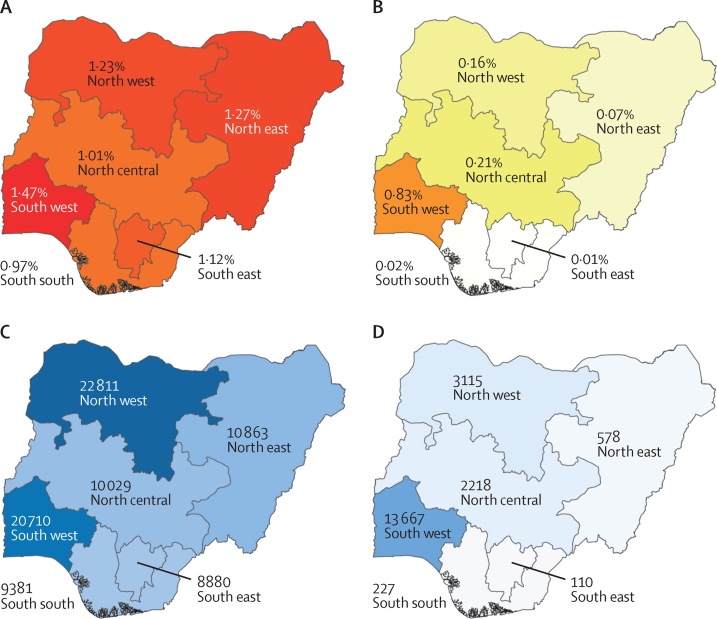
Estimated prevalence and annual births of children with *HbSS* or *HbSC* in Nigeria The estimated birth prevalence of *HbSS* (A) and *HbSC* (B) by zone and the estimated number of children born with (C) *HbSS* and (D) *HbSC* in 2015.

Assuming Hardy-Weinberg equilibrium, we estimated a national average birth prevalence of 1·21% (95% CI 1·09–1·37) for *HbSS* and 0·24% (0·19–0·31) for *HbSC*. This would have resulted in about 83 100 births with *HbSS* (95% CI 74 700–94 100), 17 700 births with *HbSC* (14 100–22 600), and 100 700 births with sickle cell disease (90 000–115 000) across Nigeria in 2015 (figure 2).

**Figure 3:**
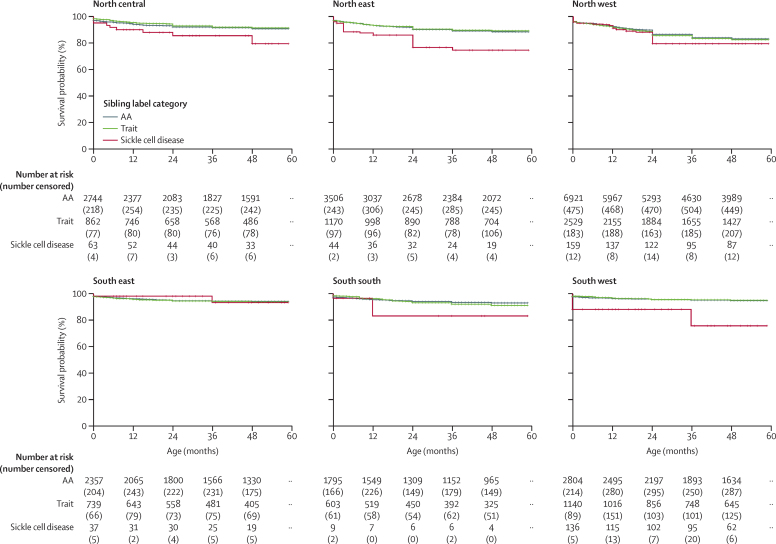
Survival curves for tested children and their untested siblings in Nigeria's 2018 Demographic and Health Survey Data are shown for Nigeria's six geopolitical zones for the three sibling-label categories AA, Trait, and Sickle cell disease. Children born 0–14 years before the survey were included.

Because we could not measure the mortality of tested children, who were by definition alive during the survey, we studied the mortality of their siblings using birth histories (figure 1). Of 11 234 tested children, 10 195 could be matched to the individual mother survey, including 89 children with *HbSS* and 29 with *HbSC*. More than a third of 1–4-year-olds, living or dead, were tested or had siblings who were tested (appendix p 8). Overall, the matched tested children had 17 205 identified untested siblings born within the previous 15 years, for a total of 27 400 children in the matched survival modelling dataset. These children were assigned to SLCs as described in the Methods. After applying sample weights, there were an equivalent of 27  619 children, of whom 449 (1·6%) were in the Sickle cell disease SLC (ie, children who tested positive for *HbSS* or *HbSC* and their full siblings), 7043 (25·5%) in the Trait SLC (children with *HbAS* or *HbAC* and their full siblings, as well as half-siblings of children with *HbSS* or *HbSC* not already assigned to the Sickle cell disease SLC), and 20 127 (72·9%) in the AA SLC. Among children born 5–14 years before the survey, the SLC distribution was 241 (1·6%), 3816 (24·8%), and 11 322 (73·6%) in the Sickle cell disease, Trait, and AA SLCs, respectively, after weighting (table 2). Of 449 weighted children in the Sickle cell disease SLC, 85 (18·8%) had died before age 5 years, compared with 722 (10·3%) of 7043 and 2041 (10·1%) of 20 127 in the Trait and AA SLCs, respectively. Of 15 378 children born 5–14 years before the survey, weighted crude under-5 mortality rates were 22·0% (53 of 241), 13·8% (525 of 3816), and 12·8% (1449 of 11322) for the Sickle cell disease, Trait, and AA SLCs, respectively. Weighted, non-parametric, Kaplan-Meier survival curves for the three SLCs, calculated separately by zone, are shown in figure 3 and as a single national curve in the appendix (p 10).

**Table 2:** Estimated composition of each sibling-group label category for children born 5–14 years before the survey

	**Overall sample weight**	**Estimated *HbAA***	**Estimated trait (*HbAS* plus *HbAC*)**	**Estimated *HbSS* plus *HbSC***	**Estimated contribution to the population with sickle cell disease**
AA SLC	73·6%	89·3% (88·3–90·3)	10·4% (9·5–11·3)	0·33% (0·23–0·43)	17·2% (12·0–23·0)
Trait SLC	24·8%	44·7% (42·5–46·9)	52·4% (50·0–54·7)	2·9% (2·4–3·6)	51·6% (44·8–58·5)
Sickle cell disease SLC	1·6%	22·1% (16·6–30·0)	49·9% (42·1–57·3)	28·1% (21·7–35·6)	31·1% (24·5–37·7)

Data are % or % (95% credible intervals). The middle three columns form the composition matrix M referred to in the Methods. SLC=sibling-group label category.

Using Cox regression pooling all zones together and stratifying by zone and urbanicity, children in the Sickle cell disease SLC born during the 15 years before the survey (both tested and untested) had an under-5 mortality hazard probability that was twice that of those in the AA SLC (hazard ratio [HR] 2·0, 95% CI 1·2–3·3; p=0·011). Children in the Trait SLC had an under-5 mortality hazard probability equivalent to that of children in the AA SLC (HR 1·0, 95% CI 0·9–1·2; p=0·76). Linear estimates of excess mortality among siblings born 5–14 years before the survey showed about 99 excess deaths per 1000 livebirths (95% CI 55 to 144; p<0·0001) in the Sickle cell disease SLC compared with the AA SLC. The Trait SLC had a non-significantly higher mortality of nine excess deaths per 1000 livebirths (95% CI −4 to 22; p=0·17) than the AA SLC.

The results showed that having a sibling with sickle cell disease in the tested sample (ie, the Sickle cell disease SLC) was associated with higher child mortality, which we assumed was predominantly due to their higher probability of having sickle cell disease themselves. By deducing the average genotype composition of each SLC, we estimated the average mortality associated with the actual genotypes themselves (figure 1). The expected underlying genetic composition of each SLC among children born 5–14 years before the survey, estimated as detailed in the Methods, is shown in table 2. Despite the higher probability of having sickle cell disease among siblings in the Sickle cell disease SLC, because of the much larger size of the other two categories, 51·6% (95% CI 44·8–58·5) of the overall population with sickle cell disease is expected to be in the Trait SLC, 17·2% (95% CI 12·0–23·0) in the AA SLC, and 31·1% (95% CI 24·5–37·7) in the Sickle cell disease SLC (table 2).

Assuming that all children with sickle cell disease had on average the same mortality risk, regardless of which sibling-group label category they were assigned to, and likewise for all genotypes, we estimated that the group of children with sickle cell disease born 5–14 years before the survey (with at least one younger sibling in the survey) had about 370 excess under-5 deaths per 1000 livebirths (95% CI 150 to 580; p=0·0008) than children with *HbAA*. Children with sickle trait had equivalent mortality to those with *HbAA* (two fewer deaths per 1000 livebirths, 95% CI −62 to 58; p=0·95). The estimated national average under-5 mortality from sickle cell disease for this cohort of siblings was 490 per 1000 livebirths (95% CI 270–700), 4·0 times higher (95% CI 2·1–6·0) than children without sickle cell disease. Nigerian children with sickle cell disease born between 2003 and 2013 with a living sibling born between 2013 and 2018 contributed an estimated 5·6% (95% CI 3·1–8·3) of the national under-5 mortality burden, and their excess mortality attributed to sickle cell disease was 4·2% (95% CI 1·7–6·9) of the national under-5 mortality. The estimated weighted average under-5 mortality across all subgroups was 127 per 1000 livebirths.

Applying UNICEF estimates of child deaths for the relevant time period, these results suggest that there were approximately 35 000 excess under-5 deaths per year (95% CI 15 000–55 000) attributable to sickle cell disease in Nigeria. Extrapolating to the entire malaria belt region, excess mortality from sickle cell disease from the mid 2000s to the mid 2010s was around 100 000 under-5 deaths per year; this is a rough order-of-magnitude assessment, since we do not have analogous survey data from other countries.

Sensitivity analyses, as well as mortality estimates using more traditional methods^7^ are in the appendix (pp 2–5 and 5–6, respectively).

## Discussion

We used data from the first DHS to test for sickle cell disease to estimate the national and subnational prevalence of disease in Nigeria, then matched data on sickle cell disease status with birth history data to estimate under-5 mortality associated with sickle cell disease. The national birth prevalence of sickle cell disease (assuming Hardy-Weinberg equilibrium) was nearly 1·5% with 0·24% (95% CI 0·19–0·31) attributable to *HbSC*, consistent with previous estimates.^20^, ^21^ Piel and colleagues^20^ estimated an *HbSS* birth prevalence of 1·4% (IQR 0·1–2·9) in Nigeria. Modell^21^ estimated a conception prevalence of 1·6% for *HbSS* and 0·5% for *HbSC*. In our study, each geopolitical zone in Nigeria had an *HbSS* birth prevalence of 1% or higher, whereas *C* allele frequencies were highest in the south-west region and relatively rare in the eastern half of the country. Previous prevalence estimates were hampered by irregular geographical coverage, and often relied on data from neighbouring countries.^22^ The 2018 DHS included samples from every state in Nigeria, revealing an *S* allele hotspot in the north-east region that had not been seen in previous maps. We also found that some parts of the south-west region had nearly equal numbers of *HbSC* and *HbSS* births.

Although the DHS provided genotype information only for living children, we identified sibling groups and used an approach to estimate under-5 mortality associated with sickle cell disease among their older siblings. Our estimates suggest a child mortality burden from sickle cell disease in Nigeria that is closer to the higher range previously reported (5–10% of overall child mortality) than to the lower estimates reported in the GBD study. Another analysis of the Nigeria DHS^23^ estimated the odds of dying at any age as being 2·5 times higher (p<0·001) among siblings of children with sickle cell disease versus siblings of other children, consistent with our sibling-group label category estimates. This other analysis did not include the additional step of estimating genotype-associated mortality.

Few studies have quantified excess mortality associated with sickle cell disease in sub-Saharan Africa. Our estimates are in general agreement with a large cohort study in Kilifi, Kenya,^6^ which showed that children with sickle cell disease had 58 deaths per 1000 person-years before age 5 years. Although this rate is half of our estimate for Nigeria, it was 23 times higher than under-5 mortality of children in that cohort without sickle cell disease. Most deaths associated with sickle cell disease in the Kilifi study took place among those who did not enter the study's treatment programme; this subgroup had about 50% under-5 mortality. The observed subnational patterns in Nigeria, although of insufficient sickle cell disease sample size to draw inference, also suggest that reductions in overall child mortality do not necessarily translate into substantial improvement in survival from sickle cell disease. A hospital-based prospective cohort study in Tanzania,^24^ at a time when penicillin prophylaxis was not part of the standard of care for patients with sickle cell disease, found that mortality was 73 per 1000 person-years for children aged under 5 years.

Our study had limitations. The absence of children under 6 months old from the sample might have caused an under-estimation of sickle cell disease prevalence and child mortality. The point-of-care tests are not perfectly accurate; the DHS reported 85% sensitivity and 91% positive predictive value.^12^ These numbers suggest that the burden is more likely under-estimated rather than over-estimated. Additionally, the DHS did not test for other conditions related to sickle cell disease such as beta thalassaemia, important modifiers of disease such as the proportion of fetal haemoglobin, or the presence of other gene variants.^2^, ^25^ Our assumption of Hardy-Weinberg equilibrium ignores the possibility of high consanguinity, which could increase the prevalence of sickle cell disease.^18^ The potential survival advantage of children with sickle trait (*HbAS*) where the malaria burden is high^2^ could elevate the observed prevalence of the *S* allele and lead to an overestimation of the expected prevalence of *HbSS*. Despite these potentially large departures from the Hardy-Weinberg equilibrium assumptions, the observed *HbSS* prevalence among children assuming Hardy-Weinberg equilibrium at the national, regional, and state levels appear reasonably self-consistent (appendix p 9).

Our mortality estimates represent children born between 2003 and 2013 with living siblings aged 6–59 months during the survey, and whose data could be matched between the household and maternal birth-history modules. Generally, this group should be somewhat better off than the entire birth cohort. Indeed, our data indicate 127 deaths per 1000 livebirths across all genotypes, whereas an age-weighted average of these cohorts' official under-5 mortality^12^ yielded 131 deaths per 1000 livebirths. The identification of full siblings in our analyses assumed that children in the same household had the same biological father. As we did not have substantial evidence to the contrary, we assumed that this was usually true. To estimate the posterior probabilities of parents and siblings we had to make assumptions on relative survival with sickle cell disease to parenthood (offset in terms of allele balance by a somewhat higher prevalence of parents with sickle trait). Our choice of a random variable centred at 50% is plausible and potentially conservative; sensitivity analyses showed that our estimate of the sickle cell disease mortality burden is robust to changes in this assumption (appendix p 3).

The small sample size of children found with sickle cell disease led to relatively wide CIs of mortality estimates, and prevents more detailed analysis at the state level or of other demographic variations in child mortality, separating *HbAS, HbAC, HbSS*, and *HbSC* for the main analysis, accounting for health-system variations, or examining regression interactions. We therefore recommend testing the entire sample for sickle cell disease in future surveys that undertake a similar endeavour to enable more detailed analyses. Prospective longitudinal cohort studies of patients with sickle cell disease might be the best way to study the risk factors of poor outcome and effectiveness of different interventions;^5^, ^24^, ^26^, ^27^ conversely, random cross-sectional surveys have the advantage of better representing the population.

Sickle cell disease has received far less continental and global attention and funding in sub-Saharan Africa than malaria, HIV, and vaccine-preventable diseases. The mortality estimate reported here would make sickle cell disease Nigeria's sixth-largest cause of child death in the GBD, after diarrhoea, malaria, lower respiratory infections, preterm birth, and birth asphyxia. High-prevalence countries might find it hard to meet the Sustainable Development Goal of fewer than 25 under-5 deaths per 1000 livebirths by 2030, until child mortality and morbidity from sickle cell disease are addressed. Straightforward and affordable public-health solutions for management of the disease have become more readily available for deployment.^28^ The challenge is to implement them equitably on a large scale across sub-Saharan Africa.

## Data sharing

The de-identified individual-level data used in this study can be obtained from the Demographic and Health Survey programme by registered users upon approval of a study description. Instructions for obtaining these data are at https://dhsprogram.com/.

## Declaration of interests

OEN is the primary investigator of the Sickle Pan African Research Consortium in Nigeria and acknowledges support from the National Heart, Lung, and Blood Institute for the Sickle Pan African Research Consortium. APO and DLC are employed by the Institute for Disease Modeling, a research group within, and solely funded by, the Bill & Melinda Gates Foundation. FBP is a member of the UK Medical Research Council (MRC) Centre for Environment & Health, funded by the UK MRC, and of the Health Protection Research Unit in Chemical and Radiation Threats and Hazards, a partnership between Public Health England and Imperial College London, which is funded by the National Institute for Health Research (NIHR). FBP also acknowledges infrastructure support for the Department of Epidemiology and Biostatistics provided by the NIHR Imperial Biomedical Research Centre, and received fees from bluebird bio for work unrelated to this study. All other authors declare no competing interests.
