# Supplementary material for: Child mortality from sickle cell disease in Nigeria: a model-estimated, population-level analysis of data from the 2018 Demographic and Health Survey
Source: Lancet Haematol. 2021 Sep 2;8(10):e723–31. doi: 10.1016/S2352-3026(21)00216-7 (PMC8460996; doi:10.1016/S2352-3026(21)00216-7)
Supplement: Supplementary appendix [file mmc1.pdf]

THE LANCET
Haematology

Supplementary appendix

This appendix formed part of the original submission and has been peer reviewed. We post it as supplied by the authors.

Supplement to: Nnodu OE, Oron AP, Sopekan A, Akaba GO, Piel FB, Chao DL. Child mortality from sickle cell disease in Nigeria: a model-estimated, population-level analysis of data from the 2018 Demographic and Health Survey. *Lancet Haematol* 2021; published online Sept 2. https://doi.org/10.1016/S2352-3026(21)00216-7.

Methods S1: Decomposing SLC Models for Genotype Mortality Estimates 2
Methods S2: Sensitivity Models at the SLC Level 2
Methods S3: Additional Sensitivity Analyses 3
Sensitivity to parental SCD frequency 3
Simulations to Examine the Decomposition Method 3
Logistic Regression Version 4
Indirect "Spillover" Mortality Effect on Siblings 4
Tested Children with Age over 5 Years in Maternal Interview 4
Methods S4: Cross-sectional methods for estimating mortality 5
Methods 5
Results 5
Supplementary Figures 7
Figure S1. S allele frequency by strata. The S allele frequencies among urban or rural populations by state. 7
Figure S2. The proportion of children in the child survival dataset (individual mother interview), who were tested, or had a sibling tested for HBB in the Nigeria 2018 DHS. Circle area is proportional to sample size. 8
Figure S3. Expected vs observed frequency of HbSS in Nigeria. The observed frequency of children in the Nigeria 2018 DHS at the national, regional, at state level are plotted on the y-axis. The frequency expected under Hardy-Weinberg equilibrium assumptions are plotted on the x-axis. The expected HbSS frequency was the S allele frequencies at the national, regional, and state levels squared. The gray dotted line is where observed equals expected. 9
Figure S4. Kaplan-Meier survival curves for tested children and their siblings (by SLC) in Nigeria's 2018 DHS. Children born 0-14 years prior to the survey are included. 10
Supplementary Table 11
Table S1. Hb genotype results from the 2018 Nigeria DHS. For the right column, household sample weights were applied. Age ineligible and non-de facto residents were excluded. 11

Supplementary Methods and Sensitivity Analyses

Methods S1: Decomposing SLC Models for Genotype Mortality Estimates

Our overall prevalence estimates (using the entire tested dataset) were used to provide a prior distribution of parent genotypes for each of 12 demographic subgroups defined by the model (zone and urban/rural). In these calculations we assumed that SCD genotypes survive to parenthood, on average, half as often as non-sickle genotypes, and adjusted trait (HbAS and HbAC) prevalence among parents to match the observed allele prevalence among children in each model stratum. We also made the simplifying assumption that parent mating is homogeneous within each demographic subgroup, unaffected by local community preference or genetic consulting efforts, due to the paucity of reliable systematic data regarding this question.

We summed the weighted posterior sibling distributions within each sibling label category (SLC) across all households and demographic subgroups, producing a 3x3 expected composition matrix, *M* (**Figure 1e**). All posterior calculations excluded the rare HbCC genotype. Based on the latent-variable assumption described above, the relationship between SLC model effects and true genotype group effects becomes a simple set of linear equations:

$$Equation1: M\gamma = \beta,$$

where β is the vector of 3 excess SLC mortalities (β_1 representing the reference “AA SLC” is zero by definition), and γ the underlying excess genotype mortalities. An estimate for γ can therefore be found by simply inverting *M*, then realigning so that HbAA survival is the reference (**Figure 1f**):

$$Equation2: \hat{\gamma} = M^{-1}\hat{\beta} - M^{-1}\hat{\beta}_1$$

We estimated uncertainty by propagating model uncertainties in the multiplication and subtraction in **Equation 2**, as well as imputing the composition matrix 1000 times using the bootstrap prevalence estimates, with relative SCD survival to parenthood randomly drawn from a Beta(3,3) distribution, in such a way that in each of the 1000 realizations it is a single constant rate, whose average across realizations is 0.5. The imputed effect estimates were combined into overall point estimates and standard errors using standard multiple-imputation methodology [23].

Secondary and sensitivity analyses included a Cox proportional hazards regression to estimate the SLC variable's effect on time-censored U5M for the entire sample of tested children and siblings born 0-14 years pre-survey, and (separately) decomposition process assuming equal survival to parenthood of SCD genotypes. Additional sensitivity analyses are described in the following sections.

Methods S2: Sensitivity Models at the SLC Level

We estimated additional linear models for the 5-14 year sibling group at the category-label level: a model with all 5 genotypes, an unadjusted model (except for cluster), and a model that adjusts for selected additional standard risk factors known to affect child survival in Nigeria, and a model that excludes half-siblings of tested children.

The main model used in decomposition analysis is in the first row of each table. All models had a random intercept for survey cluster. The reference is the "AA SLC". The "extra adjusted" model adjusted for additional standard risk factors: birth year, mother younger than 18 years, birth interval <2 years, multiple birth, and polygamy.

SCD SLC Effect	Excess Deaths per 1000 Live Births	95% CI	p-value
Main Model (zone+urban)	99.4	(54.8,144.1)	<0.0001
Unadjusted	102.0	(56.8,147.2)	<0.0001

Extra Adjusted	97.0	(52.8,141.1)	<0.0001
No Half-Siblings	97.4	(53.0,141.7)	<0.0001

**Trait SLC Effect**	**Excess Deaths per 1000 Live Births**	**95% CI**	**p-value**
Main Model (zone+urban)	8.9	(-3.9,21.6)	0.17
Unadjusted	9.8	(-3.1,22.7)	0.14
Extra Adjusted	9.4	(-3.2,22.0)	0.14
No Half-Siblings	8.8	(-4.3,21.9)	0.19

A model with the same terms as the main model, but further subdividing the trait and SCD SLCs:

**SLC Effect**	**Excess Deaths per 1000 Live Births**	**95% CI**	**p-value**
"AC"	4.6	(-38.5,47.7)	0.83
"AS"	9.2	(-3.9,22.3)	0.17
"SC"	131.4	(52.3,210.6)	0.0011
"SS"	84.7	(30.9,138.5)	0.0020

Methods S3: Additional Sensitivity Analyses

Sensitivity to parental SCD frequency

Sensitivity analysis assuming equal SCD survival to parenthood yielded estimates of 320 (95% CI: 150-500) excess deaths per 1000 live births, and a contribution of 3.7% (95% CI: 1.2%-6.4%) to the national U5M burden; an impact about 10% smaller than per our main analysis.

Simulations to Examine the Decomposition Method

We examined our novel approach on synthetic data of structure and size patterned after the DHS dataset, with surviving children 6-59m tested and their siblings matched. Specifically, we assumed a true uniform additive effect in mortality rate (compatible with linear regression) or a true uniform increase in mortality odds (compatible with logistic regression). We identified SLCs, fitted SLC regression models, then applied the decomposition to estimate genotype effect, repeating the process 1000 times for each setting, and repeating again using a misspecified model (i.e., linear regression with a mortality-odds increase and vice versa). We found that linear regression performed better both when it was correctly specified and misspecified. It was unbiased, produced less variable estimates, and detected a statistically significant effect more often than logistic regression. The latter also returned effect estimates of the correct order of magnitude, but tended to have an upward overall bias, at least in part due to exponentiation. After adding a downward exponentiation correction and the conservative assumption that all genotypes survive to parenthood equally, the median of logistic regression simulations was a few percent below the true effect, and the mean 15%-20% above it. On balance, based on these results we decided that linear regression would be more appropriate as the main analysis, even though it is not the most common choice for binary data.

Logistic Regression Version

The logistic regression version on the observed data finds that among older siblings born 5-14 years pre-survey the mortality odds of "SCD SLC" were 2.1 (1.5,3.0; $p<0.0001$) times higher than the "AA SLC", and the "Trait SLC" 1.1 (1.0,1.2;$p=0.16$) times higher. After decomposition and bias mitigation, the resulting point estimates of population burden were still somewhat higher than linear regression (510 vs. 480 SCD deaths per 1000 live births; 4.8% excess-mortality contribution to the national burden vs. 4.2%), albeit with broader confidence intervals ([230,780] and [1.3%,8.0%], respectively). Notably, the logistic-regression generated CIs completely contain the linear-regression CIs reported in the main results.

Indirect "Spillover" Mortality Effect on Siblings

There may be reason to believe that rather than driven only by genotype directly, some of the SLC effect may be indirect, i.e., on non-SCD siblings of children with SCD. It has been reported that families with SCD children suffer disproportionate financial and emotional burden, which may impact survival of all their children. We explored a simple sensitivity model of the impact of such an indirect effect, assuming that such siblings suffer a proportion of the SCD effect. This is equivalent to shifting some of the non-SCD prevalences into the SCD column in the composition matrix M, and then performing the decomposition as in the main model. We implemented such a decomposition, assuming the relative indirect effect is random uniform between 0 and 40% of the SCD effect (mean 20%), and that on average 2 siblings are close enough to the child with SCD (or are younger) to experience the effect. The effect size was informed by the approximate effect of falling one quintile down in Nigeria's socioeconomic quintiles according to the DHS.

The resulting SCD U5M estimate decreased to 380 (95% CI:220,530) per 1000 live births, and the direct excess SCD mortality contribution to the national burden, to 3.0% (95% CI:1.2%,5.0%). However, we must add the indirect effect felt by non-SCD siblings, since it is part and parcel of the impact of under-treated SCD in the community. The indirect effect increases the overall impact of SCD on the national U5M burden to 3.7% (95% CI:1.4%,6.2%) - not far below the impact estimate without assuming indirect effects. In any case, barring solid quantitative evidence regarding the extent of indirect mortality effect, this assumption is unidentifiable from survey data alone.

Tested Children with Age over 5 Years in Maternal Interview

Lastly, as described in Methods children qualified for the test if they were 6-59 months old in the household (HH) module. However, the determining age for survival analysis is in the maternal interview module; arguably the more reliable age entry. Three children in the tested and matched sample were listed as over 5 years old in the maternal interview.

One child was 59 months old in the HH module and 60 in the maternal interview. They were the only tested child in their sibling group. Since the two modules were not necessarily simultaneously collected, this does not represent a genuine discrepancy. To be compatible with the rest of the data, the 59/60 month old child was excluded from the main 5-14 years survival analysis, but their older siblings were included using the tested child's genotype to determine their label.

A greater challenge was posed by the remaining two children's data, both coming from HHs with multiple tests. Both had greater discrepancies between HH and maternal-interview ages, and in both cases the latter indeed seems more reliable (both had siblings within <9 months of age in the HH module). Unfortunately, in one HH the tested child in question had SCD, altering their sibling-group's label. It was a rather large HH with relatively high survey weight, enough to affect model estimates visibly. Excluding test information for these two children while including them in the analysis due to their older age, which may be seen as most self-consistent analysis approach, changes the label of one HH and causes SCD effect estimates to go up by ~8%: the "SCD SLC" point estimate increases from 99 to 107 per 1000 births, the decomposed SCD estimate increases from 360 to 390 per 1000, and the excess-mortality attribution increases from 4.2% to 4.5% of Nigeria's national U5M burden. Confidence intervals shift accordingly.

To be conservative, we chose the analysis as reported in the main article despite its slight inconsistency. This illustrates however that the precision of future SCD burden estimates would benefit greatly from testing the entire age-eligible HH sample rather than only one-third, and possibly also from lowering the eligibility age, since manufacturers of both widely available POCT kits guarantee their reliability at ages substantially younger than 6 months.

Methods S4: Cross-sectional methods for estimating mortality

Methods

By definition, only children alive during the survey were typed for HBB. We used two known approaches and one novel one to work around this information constraint and obtain survival estimates. Due to the relatively small sample of tested children with SCD, most of our inference for SCD-associated survival pools both SCD genotypes and all zones together. We examined the HbSS genotype alone as secondary analysis.

The age pyramid approach compares the SCD proportion among younger and older children. Clinical knowledge suggests that during the first few months after birth, SCD infants still survive as well as other genotypes. Therefore, the best reference group is newborns or infants (Grosse et al. 2011). The closest approximation to this in our dataset are tested infants 6-11 months old. Therefore, we compared the SCD proportion among them and among older tested infants. The survival ratio between sickle (S) and other (A) children is

$$\text{Equation S1: } \frac{1-q_S^t}{1-q_A^t} = \frac{r^t}{r^0},$$

Where the superscripts *0,t* denote the reference and older age intervals, respectively, *r* is the ratio between the surviving SCD and non-SCD counts, and q^t indicates the average raw death proportion between the two intervals. All proportions are weighted by the survey's sampling weights.

A second approach to estimating mortality is deficit in children with SCD, compared with the number predicted from Hardy-Weinberg equilibrium (HWE) between HbAA and sickle-trait (HbAS or HbAC) counts. Given the large subnational disparity in prevalence, HWE is calculated separately by the six geopolitical regions. The overall survival ratio is

$$\text{Equation S2: } \frac{1-q_S}{1-q} = \frac{p_S}{p_S^{HWE}},$$

Where *p* indicates the SCD proportion, and *q* with no subscript or superscript is the population proportion of children born 6-59 months ago (out of the entire DHS sample) who had died before the survey, across all genotypes. Here too we use sample weights. In principle if the observed SCD proportion is substantially higher than the HWE, one may encounter negative mortality estimates. Note that using HWE is conservative, i.e., favorable towards SCD survival, since assortative mating leads to above-HWE proportions of minority genotypes.

For both approaches above, our bootstrap prevalence draws were used to estimate the uncertainty in survival ratios. We also incorporated Normal-approximation uncertainty to the population-average survival estimates, based on the relevant DHS sub-sample size.

Results

The weighted ratio between HbSS and HbAA/HbAS among tested infants (6-11m old) was 0.012, compared to 0.0087 among 12-59m; a 28% relative decrease. Conversely, HbSC proportions vs. HbAA/HbAC were somewhat higher among the older children. Therefore, the overall ratio between all SCD and non-SCD genotypes was only 13% lower among 12-59m old than among infants. Bootstrap results indicated high variability and lack of statistical significance, due to small SCD counts among infants: 13% of bootstrap runs yielded an increase rather than a decrease in HbSS proportion between 6-11m and 12-59m, as did 31% of runs for all SCD genotypes combined.

Comparing the SCD proportion among the entire 6-59m tested sample (excluding "other") to Hardy-Weinberg equilibrium (HWE) estimates that use only HbAA and trait proportions, similar trends are observed but with somewhat stronger significance: there was a 14% (-4%,34%) HWE deficit among all SCD, and a larger and statistically significant 27% (8%,46%) deficit among HbSS only. Using population-average cross-sectional mortality from the entire maternal-interview sample (10.2%), we obtain estimates of 2.3 (0.6,4.3) and 3.6 (1.7,5.6) times higher mortality among children with SCD and HbSS, respectively, compared with all other children. This translates into excess-mortality attribution estimates of 1.9% (-0.5%,5.1%) and 3.1% (0.8%,5.8%) out of the national mortality burden (of children who would have been ages 6-59m at the time of the survey?), to SCD and HbSS, respectively. The ordering of the latter point estimates is unrealistic, of course, since the SCD group includes HbSS, but there is substantial overlap between the confidence intervals. Note that this specific analysis does not describe under-5 mortality, but rather the cross-sectional survival proportion among children born 6-59m (median 32 months) before the survey.

References

1. Piel FB, Adamkiewicz TV, Amendah D, Williams TN, Gupta S, Grosse SD. Observed and expected frequencies of structural hemoglobin variants in newborn screening surveys in Africa and the Middle East: deviations form Hardy-Weinberg equilibrium. Genet Med. 2016 Mar;18(3):265-74. doi: 10.1038/gim.2015.143.

2. Chen JJ. The Hardy–Weinberg principle and its applications in modern population genetics. Front Biol. 2010; 5:348–353. doi: 10:1038/gim.2015.143.

3. Dong Y, Peng CY. Principled missing data methods for researchers. *Springerplus*. 2013;2(1):222. doi:10.1186/2193-1801-2-222

Supplementary Figures

Figure S1. *S allele frequency by strata. The S allele frequencies among urban or rural populations by state.*

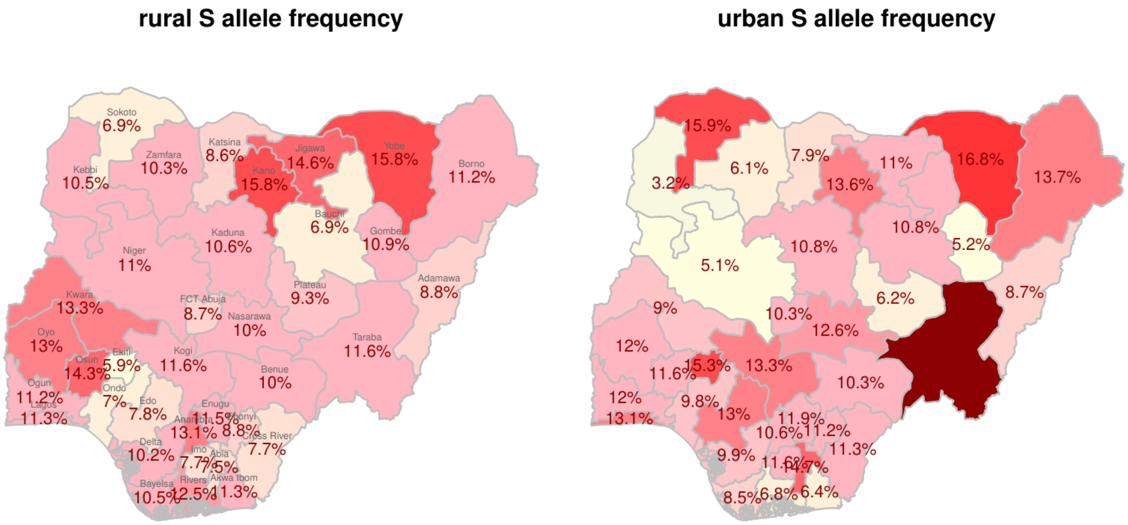

Figure S2. *The proportion of children in the child survival dataset (individual mother interview), who were tested, or had a sibling tested for HBB in the Nigeria 2018 DHS. Circle area is proportional to sample size.*

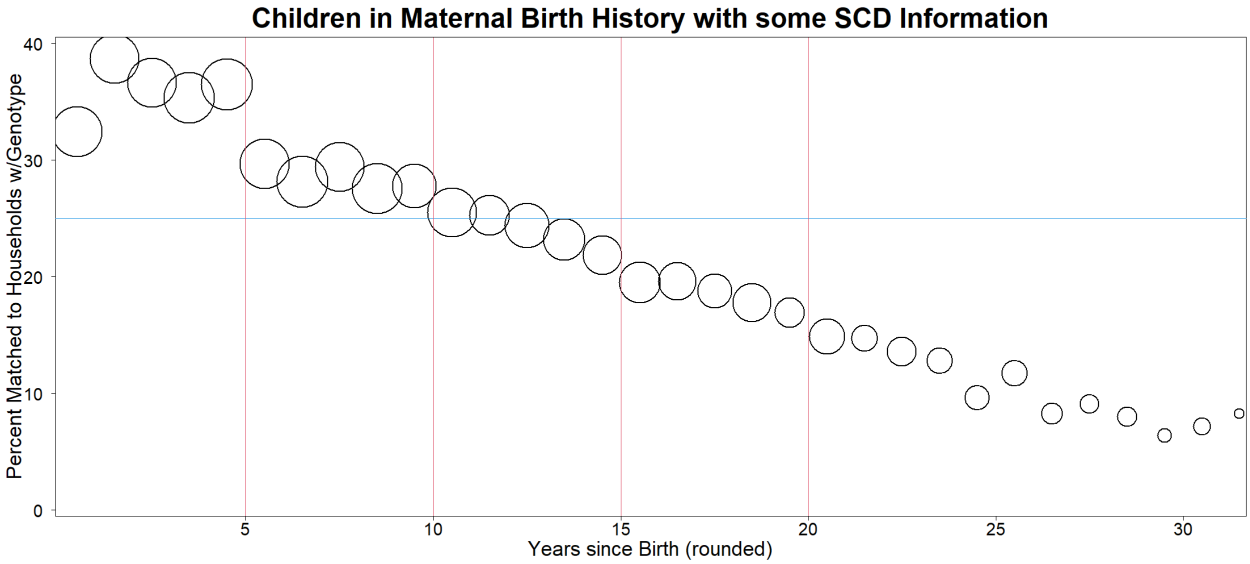

Figure S3. *Expected vs observed frequency of HbSS in Nigeria. The observed frequency of children in the Nigeria 2018 DHS at the national, regional, at state level are plotted on the y-axis. The frequency expected under Hardy-Weinberg equilibrium assumptions are plotted on the x-axis. The expected HbSS frequency was the S allele frequencies at the national, regional, and state levels squared. The gray dotted line is where observed equals expected.*

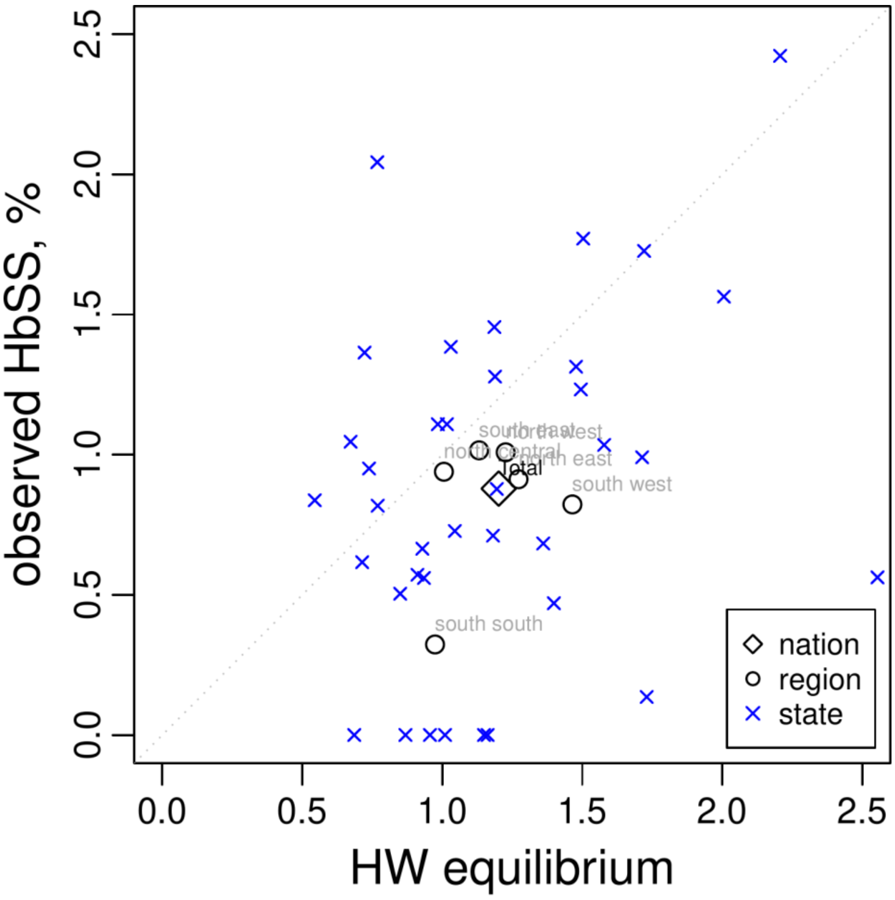

Figure S4. *Kaplan-Meier survival curves for tested children and their siblings (by SLC) in Nigeria's 2018 DHS. Children born 0-14 years prior to the survey are included.*

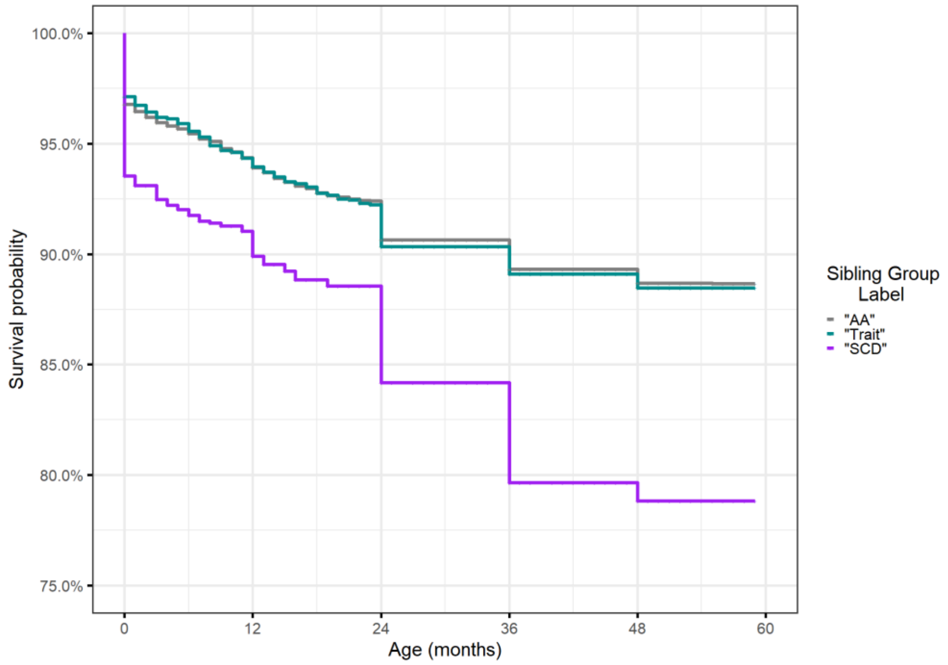

Supplementary Table

Table S1. *Hb genotype results from the 2018 Nigeria DHS. For the right column, household sample weights were applied. Age ineligible and non-de facto residents were excluded.*

Genotype	***Count***	***Percent***	***Weighted percent***
AA	8,700	77.8%	77.2%
AS	2,186	19.5%	19.7%
AC	155	1.4%	1.6%
SC	34	0.3%	0.4%
SS	102	0.9%	0.9%
“Other”	9	0.1%	0.1%
Total	11,186	100.0%	100.0%
